# Hormone therapy outcome in lymphedema

**DOI:** 10.18632/aging.101772

**Published:** 2019-01-16

**Authors:** Barbara Garmy-Susini

**Affiliations:** 1 I2MC, INSERM UMR 1048, CHU-Rangueil, Université de Toulouse, Toulouse, France

**Keywords:** lymphedema, hormone therapy, estrogen, tamoxifen, menopause

The lymphatic system drains interstitial fluids, fat, and immune cells through the lymph nodes and return fluids back to the blood circulation in the jugular area [1]. A dysfunction of the lymphatic system promotes lymphedema, a disorder characterized by impaired lymphatic return and swelling of the extremities by accumulation of fat and fibrosis in the arm or the leg [1,2].

Lymphedema can be an inherit condition caused by a genetic mutation (primary lymphedema) or occurs after cancer treatment or filarial infection (secondary lymphedema) [3]. In western countries, lymphedema arises predominantly after breast cancer. More than 10 percent of breast cancer survivors develop secondary lymphedema within months, sometimes years after surgery, suggesting that this pathology is not only a side effect of the surgery, but involves a contribution of cancer treatments. Despite millions of women affected by this disabling condition, the effect of hormones and in particular hormone therapy on lymphedema has been poorly investigated.

Recent paper by Morfoisse et al. explored the role of estrogens on lymphatic endothelial cells. They identified the estrogen receptor α (ERα) as a key player of the lymphatic endothelial function. In agreement with these findings, they highlighted the detrimental role of hormone therapy on the lymphatic system leading to an aggravation of lymphedema [4].

The estrogen receptor is the most important biologic marker of response to treatment in breast cancer. It is a member of the family of nuclear steroid receptors and functions as a transcriptional regulator, which is controlled by the 17β-estradiol (E2), the most prominent estrogen. ERα also mediates non-transcriptional mechanisms, called non-genomic signal associated with the activation of mitogen-activated protein kinases (MAPK) or the phosphatidylinositol 3-OH kinase (PI3K) signaling pathways [5].

There is a large body of evidence suggesting that female hormone could modulate lymphatic drainage, but the effect of estrogen on lymphatic network has been surprisingly poorly investigated. The hormonal status of patients is critical for determining suitable adjuvant treatment. Tamoxifen is the most commonly used hormone therapy in decades for premenopausal women with breast cancer [6]. It is a partial agonist of ER. After menopause, people switch to an aromatase inhibitor that blocks the conversion of testosterone to estrogens.

The study by Morfoisse et al. established the crucial role of female hormone, in particular E2, on the lymphatic endothelium. They found that the development of lymphedema, a lymphatic dysfunction in breast cancer survivors, is not only a side effect of surgery, but is highly dependent of the hormonal status. This study shows that women develop more lymphedema after hormone therapy, in particular tamoxifen, the major hormone therapy used for pre-menopausal women.

To better understand molecular mechanisms that regulate lymphedema, they developed an original mice model of unilateral lymphedema. In parallel, they processed a device based on microwave reflection properties that are modified depending on tissue water and ion content, which allow a non-invasive early detection of lymphedema.

Morfoisse and colleagues found a beneficial effect of estradiol on lymphatic function and drainage. Estradiol protects from edema and this effect is mediated by its receptor ERα, but not β, in lymphatic endothelial cells. Estradiol promoted lymphangiogenic gene activation and skin microenvironmental modifications. This effect was confirmed in Tie2-Cre; ERα^-/-^ mice in which the protective the estrogen receptor is depleted in the lymphatic endothelium.

In that context, tamoxifen abrogated estradiol-induced beneficial effects by inhibiting both genomic and non-genomic effect on lymphatic basal function. Tamoxifen also alters lymphatic endothelial shape, in particular filopodia formation, to reduce lymphatic endothelial cell migration and branching by inhibiting Akt, but not Erk phosphorylation.

This article provides the first evidence of a protective effect of the estrogens on the lymphatic system, and suggests that a defective hormonal balance generated by hormone therapies could participate to lymphedema formation in breast cancer women survivors.

**Figure 1:**
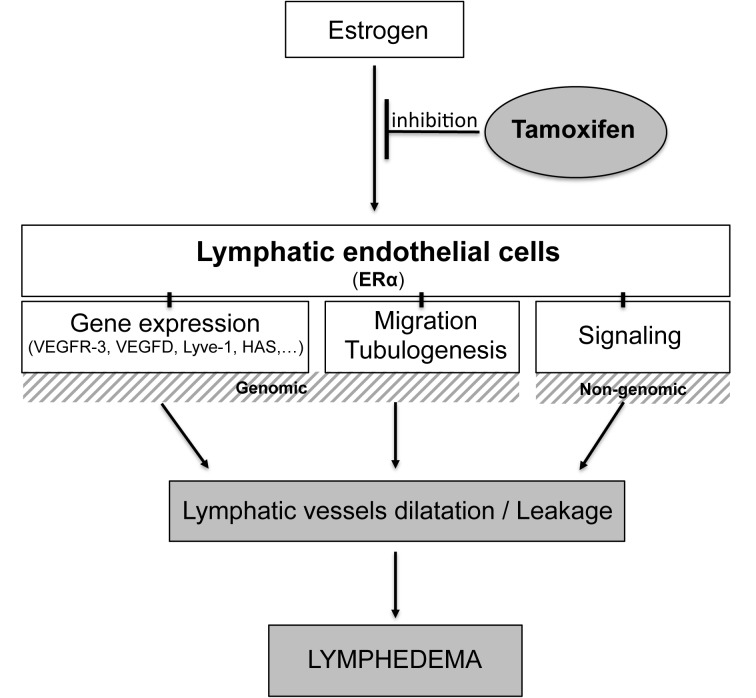
**Tamoxifen treatment leads to lymphatic dysfunction and aggravates lymphedema.** Tamoxifen inhibits estrogen binding to its receptor ERα on lymphatic endothelial cells to block both genomic and non-genomic pathways. After long-term delivery, the blockade of ERα by hormone therapy leads to lymphatic dilatation and leakage, the main features of lymphatic shape in lymphedema.

Figure 1 summarizes this recently published study showing that estrogens play a crucial role in lymphatic vessel function and protect from lymphedema. This report suggests that chronic long-term delivery of tamoxifen, has a deleterious impact on lymphatic vessel drainage and that tamoxifen affects both lymphatic endothelial cell gene expression and microenvironment.

Altogether it preconizes a better management of patients according to their hormonal status linked to menopause to prevent from lymphedema formation.
